# Strategies to Prevent Surgical Site Infections in Orthopaedic Surgery: A Literature Review

**DOI:** 10.7759/cureus.99648

**Published:** 2025-12-19

**Authors:** Abdulrahman Younes, Hani Moosavi, Rebecca E O'Neill

**Affiliations:** 1 Department of Orthopaedics and Trauma, Barts Health NHS Trust, London, GBR

**Keywords:** arthroplasty, complication of treatment, elective orthopaedic surgery, healthcare-associated infection, infection rates, orthopaedic implant-related infection, surgical site infection (ssi)

## Abstract

Surgical site infections (SSIs) are a significant cause of excess expenditure and increased morbidity and mortality when considered in the context of orthopaedic surgery, with patients often facing prolonged inpatient stays and multiple revision procedures to address this.

SSIs, though not completely avoidable, can be mitigated through a variety of pre-operative, intra-operative and post-operative strategies. This literature review aims to offer an oversight of established processes to reduce SSIs, as well as identify other measures with the potential to do this also.

## Introduction and background

Surgical site infections (SSIs) are among the most common post-operative complications of orthopaedic surgery. SSIs can be superficial at the skin level or penetrate deeper into muscle, soft tissues and implanted materials. SSIs normally develop within 30 days of an orthopaedic procedure as per definitions, but up to and including 90 days in cases involving prosthetic implants [1,2].

SSIs are associated with increases in morbidity, revision surgery, inpatient stay duration and mortality [3,4]. SSIs account for nearly 20% of all hospital-acquired infections [1,5] and affect approximately 10% of orthopaedic procedures across 129 NHS trusts in England [6]. Within orthopaedics, variance in incidence of SSIs can be influenced by patient characteristics (both demographic and co-morbid), nature of the surgery being undertaken and any prophylactic measures taken to avoid SSIs.

Across a four-year timeframe, the incidence of SSIs within total knee and total hip arthroplasty procedures equated to 0.4% and 0.5%, respectively, in the United Kingdom [7]. In the UK, the average additional cost to treat an SSI is £3,122.86 per patient [8], largely attributed to an increased inpatient stay of 6.5 days on average [9]. Comparatively, SSIs have an annual cost of around $3.3 billion ($20,000 per admission) in the United States, with an average prolonged hospital stay of 9.7 days [10,11]. Furthermore, SSIs can be additionally considered in the context of the type of contaminant, as seen in Table 1 [12].

**Table 1:** SSI classification system SSI: surgical site infection Source: [12]

Class	Type	Description	Percentage
1	Clean	Uninfected operative wound. No bacterial flora	8%
2	Clean contaminated	Wounds affecting respiratory, alimentary, genitourinary tracts under controlled conditions and without normal contaminants	22%
3	Contaminated	Open fresh accidental wounds. Spillage of viscous contents	30%
4	Dirty	Old wounds with ischemic and/or necrotic elements, or those with retained infection	40%

Preventing SSIs therefore requires a multifocal and multidisciplinary approach that includes pre-operative, intra-operative and post-operative strategies aimed at limiting the spread of infection. Based on the current evidence base, this literature review aims to provide an overview of the established and emerging strategies aimed at decreasing SSI rates in orthopaedic surgery, irrespective of level or cause of infection.

## Review

Search strategies

Database searches were carried out from the following five databases: PubMed, PubMed Central, Google Scholar, ResearchGate, and ScienceDirect. Furthermore, where appropriate, national and international clinical guidelines were also searched when pertinent to SSIs in orthopaedic surgery. Databases and clinical guidelines were searched in accordance with the following phrases to obtain maximal results and guide meaningful conclusions (Table 2).

**Table 2:** Nonexhaustive table of search terms SSI: surgical site infection

Antibiotics in orthopaedic surgery
Antibiotic timing in orthopaedic surgery
SSI prevention in orthopaedic surgery
Prophylactic antibiotics in orthopaedic surgery
Tourniquet use in orthopaedic surgery
Management of open fractures in orthopaedic surgery
Patient factors in preventing SSIs in orthopaedics
Peri-operative factors impacting SSIs in orthopaedics
Co-morbidities and SSIs in orthopaedics
Surgical attire and SSIs
Patient preparation and SSIs in orthopaedic surgery
Impact of draping on SSIs
Physiological factors and SSIs in orthopaedic surgery
Wound management and infection in orthopaedic surgery

Pre-operative strategies

Prophylactic Antibiotics

Administering prophylactic antibiotics is a well-established, evidence-backed practice to decrease the incidence of SSIs in orthopaedic surgery. Therefore, optimal timing, duration and choice of antibiotics play a crucial role in determining their efficacy.

Timing of preoperative antibiotics: Although the use of prophylactic antibiotics preoperatively significantly reduces the rate of SSIs, the timing of antibiotic administration pre-incision remains debatable. The Centres for Disease Control and Prevention’s (CDC) guidelines in 2017 recommended that antibiotics should be given within 60 minutes preceding a skin incision [1]. In cases where an antibiotic with an infusion time between one and two hours is used, administration should begin within 120 minutes preoperatively [1].

The World Health Organisation (WHO) recommendations are equivocal to those of the CDC [13]. Interestingly, a study in 2021 demonstrated that when using antibiotics with a short infusion time, there was no difference in the risk of SSIs if antibiotics were administered 0-30 minutes prior to incision or 30-60 minutes before incision [14].

In a meta-analysis [15] that included around 54,500 patients undergoing surgery, the association between timing of preoperative prophylactic antibiotics and the incidence of SSIs was assessed. The study compared the risk of SSIs when prophylactic antibiotics were administered more than two hours before incision, between one and two hours before incision, one hour or less before incision, and after incision. The study found that administering prophylactic antibiotics two hours or more before incision showed a five-fold increase in SSI risk. Furthermore, it reported a doubled rate of SSIs when antibiotics were administered after skin incision. Overall, there was no statistically significant difference in SSI incidence at any time point of antibiotic administration within two hours of knife-to-skin [15]. Another study showed no significant difference in SSI incidence when short-infusion-time antibiotics were administered 30-60 minutes vs. 0-30 minutes preoperatively [16].

Antibiotic timing and tourniquets: Surgical tourniquets, though aiding surgery by providing a bloodless field, have also been shown to decrease antibiotic efficacy when used [17,18]. The National Institute for Health and Care Excellence (NICE) recommends administering prophylactic antibiotics at the time of induction of anaesthesia and suggests antibiotics should be given earlier for operations using tourniquets [19].

Antibiotic re-dosing

Re-dosing antibiotics ensures that antibiotic concentrations are maintained during prolonged procedures. This has been shown to reduce the risk of SSIs [20]. It is suggested that if blood loss exceeds 1,500 mL intra-operatively, antibiotic re-dosing should be considered [21,22].

NICE recommends that prophylactic antibiotic re-dosing should be carried out when the length of the procedure exceeds the half-life of the antibiotic administered [19].

The Stanford Antimicrobial Safety and Sustainability Program recommends that vancomycin does not need re-dosing due to its long half-life, even with excessive intra-operative blood loss. They also recommend that cefazolin should be administered every four hours [21]. When gentamicin is administered at a dose of 5 mg/kg, re-dosing is usually not indicated [23], unless the duration of surgery exceeds eight hours, in which case re-dosing with 2.5 mg/kg is indicated [24]. Though these recommendations are substantial, it is always important to contextualise antibiotic dosing against the patient and the intended surgery, and to follow local policy to avoid propagating antibiotic resistance.

Duration of postoperative antibiotics

The most recent CDC guidelines from 2017 [2] recommend that antibiotics should not be administered after the operation has been completed, unless there is an underlying infection being treated. A systematic review and meta-analysis showed that when best practice is followed, continuation of antibiotics post-operatively confers no additional benefit in reducing the incidence of SSIs [25]. In 2008, NICE stated that post-operative antibiotic doses are not usually required but are still frequently administered. They advise that when used, they should be given at the correct dose and timing; otherwise, post-operative antibiotic efficacy is lost [26].

The 2018 International Consensus Meeting on Orthopaedic Infections concluded that patients who did not receive post-operative antibiotics after total joint arthroplasty were not at increased risk of SSI [27]. Conversely, the American Association of Hip and Knee Surgeons raised concerns about the CDC guidance on omitting post-operative antibiotics in total joint arthroplasty, recommending 24 hours of post-operative antibiotic prophylaxis [28].

Antibiotic selection

SSIs are mainly caused by bacteria, with rarer occurrences due to fungal, parasitic or viral aetiologies. *Staphylococcus aureus*, a gram-positive bacterium, has been shown to cause up to 11.38% of SSIs following orthopaedic surgery [29]. Other gram-positive bacteria, such as *Staphylococcus epidermidis*, *Streptococcus pyogenes*, and *Streptococcus pneumoniae*, are also primary causes of SSIs. Therefore, antibiotics that cover gram-positive bacteria should be administered where appropriate [30].

In the UK, the British National Formulary (BNF) produced guidelines for the use of prophylactic surgical antibiotics. For the listed procedures, the BNF advises that antibiotics should be given up to 30 minutes before incision, and re-dosing should occur in prolonged procedures or if there is excessive blood loss.

They also outline alternative antibiotic choices when allergies prevent the first-line option from being given, and where there is a high risk of methicillin-resistant *S. aureus* (MRSA). Again, it is important to note that local guidelines may vary and should be consulted.

Joint Replacement

For hip and knee arthroplasty, the American Academy of Orthopaedic Surgeons recommends the use of either a first- or second-generation cephalosporin, or a glycopeptide antibiotic [31]. The BNF recommends a single dose of intra-venous (IV) cefuroxime or IV flucloxacillin plus gentamicin. If there are allergies preventing first-line antibiotics or a high MRSA risk, a single dose of IV teicoplanin or IV vancomycin is recommended.

Open Wounds

*Pseudomonas aeruginosa*, a gram-negative rod, can cause infection in open wounds contaminated with fresh water. These wounds usually require antibiotic coverage with levofloxacin or ciprofloxacin. However, salt-water-contaminated wounds can be infected by Vibrio species and other organisms and are treated with doxycycline [32]. When a farm injury causes an open wound, contamination with faecal matter or soil may lead to infections caused by Clostridium species, which are usually treated with a penicillin antibiotic [33].

Closed Fractures

As closed fractures possess a smaller risk of contamination than open fractures, antibiotic cover with a first-generation cephalosporin usually suffices. The BNF recommends a single dose of IV cefuroxime or IV flucloxacillin. If allergies prevent first-line antibiotics or there is a high MRSA risk, IV teicoplanin or IV vancomycin is recommended.

Open Fractures

Open fractures pose a significant risk to limb integrity. Exposure to bacteria may lead to complications, including osteomyelitis, non-union and potential amputation [34]. In a study by Suzuki et al., 77% of Gustilo-Anderson type III injuries had gram-negative bacteria isolated, requiring treatment with aminoglycosides [35]. When treating open fractures, research shows that prophylactic antibiotics should be continued for 24 hours after wound closure [36].

At first debridement, the BNF recommends IV co-amoxiclav and gentamicin or IV cefuroxime, metronidazole and gentamicin. If allergy prevents first-line antibiotics, IV clindamycin and IV gentamicin are recommended. If there is a high MRSA risk, add IV teicoplanin or IV vancomycin. At the time of fracture fixation and wound closure, a single dose of IV gentamicin and teicoplanin, or IV gentamicin and vancomycin, is recommended.

Patient optimisation

Glycaemic Control

Maintaining optimal glycaemic control is essential in reducing the risk of SSIs in patients undergoing orthopaedic surgery. Uncontrolled glucose levels preoperatively can impair wound healing and immune function in elective surgeries [2]. Further studies have demonstrated that elevated peri-operative blood glucose levels are associated with impaired wound healing and increased infection risk, even in non-diabetic individuals [37].

The CDC guidelines recommend that during surgery, blood glucose should remain below 200 mg/dL [2]. This underscores the importance of peri-operative blood-glucose management. A large retrospective analysis of 4,302 patients who underwent cardiac surgery in the US concluded that, in patients with diabetes, pre-operative glycosylated haemoglobin A1c (HbA1c) levels should be targeted below 7%-8% to minimise adverse outcomes, although the precise threshold may vary depending on patient-specific factors [38].

NICE advises against the use of insulin in non-diabetic patients to optimise glycaemic control as a way to reduce SSI risk. Other UK guidelines advise that diabetic patients’ blood glucose should remain under 11 mmol/L [39,40]. Research from general surgery shows that early postoperative glucose control is associated with fewer SSIs [28]. Moreover, a 2017 study by Tarabichi et al. showed that elevated HbA1c was associated with a higher risk of peri-prosthetic joint infection (PJI) [41].

Nutritional Status

Optimising nutritional status preoperatively is critical for bolstering immune function and reducing the likelihood of SSIs. Malnutrition has been linked to delayed wound healing and increased infection rates due to compromised immune response. Screening for nutritional deficits, particularly in older or at-risk patients, can help identify candidates for targeted nutritional intervention [42]. The European Society for Clinical Nutrition and Metabolism recommends that malnourished patients receive tailored nutritional support for 7-14 days pre-operatively, whenever feasible, to improve surgical outcomes [43,44]. In a retrospective cohort study of over 175,000 patients undergoing spinal and sacral surgery, malnourished patients were at significantly higher risk of developing SSIs [45].

Smoking Cessation

Smoking is a well-documented risk factor for postoperative complications, including SSIs, which are more prevalent in smokers than non-smokers [46]. Preoperative smoking cessation has been shown to decrease the risk of surgical complications and enhance overall recovery [47]. A study of open tibial fracture repairs showed that fractures in smokers healed more slowly [48]. Current evidence indicates that even short-term cessation (four to six weeks) before surgery significantly lowers the risk of SSIs and improves wound healing [49]. Implementing structured smoking-cessation programmes and counselling can support patients in quitting and contribute to better surgical outcomes. This approach was shown to be promising over 20 years ago in elective-surgical patients by Ratner et al. [50].

Intra-operative strategies

Surgical Technique and Environment

Operating room ventilation: Some studies show laminar airflow systems to be effective at removing dust, bacteria and smoke from operating theatres, significantly reducing the risk of SSIs during orthopaedic procedures [51]. Though laminar airflow offers advantages, consistent adherence to sterile techniques and protocols is equally crucial for infection control [52]. Other papers are more doubtful about the efficacy of laminar flow, suggesting that standard ventilation with proper protocols may be equally sufficient [52]. Some studies go further, suggesting that laminar flow is costly with minimal benefit in reducing SSI incidence [53].

Surgical attire: Surgical attire is designed to act as a physical and functional barrier between the surgical team and the patient to reduce contamination [54]. Proper surgical attire reduces the risk of SSIs in orthopaedic surgery, and this is heightened by adherence to local guidelines on the use of masks and gowns [55].

The Orthopaedic Research Society (ORS) has published a consensus statement offering insights regarding the role of surgical gowns in preventing SSIs and PJIs [56]. The current literature lacks direct evidence supporting the practice of changing gowns during extended surgical procedures to mitigate these infections. Nonetheless, there are data indicating that prolonged operative times are associated with higher SSI rates due to increased contamination on various surfaces, including those of the surgical team. Given the existing evidence, the ORS refrains from endorsing or opposing the intervention, emphasising the importance of conducting operations as efficiently as safety and technique permit.

Oftentimes, operating surgeons need to leave the operating room to attend to emergency patients, either for clinical review or to consent them ahead of theatre. Studies show that wearing the same set of surgical scrubs in between cases and when going between theatre and other clinical environments is associated with a higher risk of infection [57,58].

ORS also pointed out that low-level evidence suggests disposable gowns may be more effective in preventing bacterial dispersion in the operating room. On the other hand, moderate- to low-quality evidence indicates that both disposable and reusable gowns are equally effective at preventing infections, provided they are sterile and fluid-resistant. However, due to the limited quality of evidence, further randomised controlled trials are warranted. While the findings from ORS enhance our understanding of this topic, additional research is necessary to reach a definitive conclusion regarding the impact of gown changes on SSI risk [56].

Patient Preparation

Hair removal: Historically, surgical-site hair removal was performed before entering the operating room, but it is now more common inside the operating room. Although hair removal reduces interference at wound closure, minimal benefit has been shown in reducing SSIs [59]. NICE states that hair removal should not be routinely done to reduce SSI risk; if necessary, electric clippers with a single-use head should be used, as razor use is associated with increased SSIs [19].

Skin preparation: Skin preparations such as chlorhexidine and povidone-iodine have shown significant benefits in preventing SSIs [60]. A large randomised controlled study showed that SSI rates were lower with chlorhexidine than with povidone-iodine [61]. Chlorhexidine was associated with half the rate of SSIs compared with povidone-iodine [62]. There was no significant reduction in SSI rates with adhesive drapes (such as OpSite, Smith & Nephew, London, UK) or wound protectors in other studies [63,64].

Nasal decolonisation: A systematic review conducted by the WHO evaluated the effectiveness of mupirocin ointment, with or without chlorhexidine gluconate (CHG) body wash, for decolonisation in nasal carriers of *S. aureus* undergoing surgery [65]. The findings provide robust evidence supporting peri-operative application of 2% mupirocin ointment, particularly in patients known to carry *S. aureu*s. The review suggests that a “screen-and-treat” approach is advantageous for high-risk procedures in nasal carriers [65].

This was further demonstrated by a multicentre study supporting a rapid screening approach to reduce SSIs [66]. Overall, the evidence indicates moderate-quality benefit from the use of mupirocin ointment, either alone or with CHG body wash, in reducing healthcare-associated *S. aureus* infections compared to placebo or no treatment [66].

Surgical drapes: Surgical drapes considerably decreased the risk of SSIs [67]. There has been debate over whether iodophor-impregnated drapes significantly decrease the incidence of SSIs. Some studies found them effective in all types of surgery, while others reported no effect [68,69].

A pertinent study concluded that there is no evidence of a significant difference between reusable vs. disposable drapes in minimising SSI risk in orthopaedic and spinal procedures, indicating a need for further investigation [70].

A large RCT found that 12.0% of incisions covered with adhesive drapes tested positive for bacterial colonisation, compared with 27.4% without drapes [71]. After adjusting for preoperative colonisation and other variables, patients not using adhesive drapes had a markedly higher likelihood of bacterial contamination at skin closure and subsequent culture points. Notably, bacterial counts at the skin were exceptionally elevated in some patients without adhesive drapes, suggesting an increased risk of SSIs or PJIs if an implant had been placed. These findings underscore the potential protective benefits of adhesive drapes in reducing bacterial colonisation during orthopaedic surgery and highlight the need for further examination of their role in infection prevention.

Wound irrigation: Wound irrigation plays a critical role in both the prevention and management of SSIs. This process encompasses three key components: the method of delivery, the volume of irrigation and the type of solution used, which may include various additives. Wound irrigation has been shown to reduce necrotic debris and infectious agents from wound surfaces. Necrotic debris complicates wound closure and increases SSI risk by providing an ideal environment for pathogens [72,73].

In orthopaedic surgery, pulsed lavage, which is pressurised irrigation, is often used [61]. Although wound irrigation has not consistently shown benefit in reducing SSI rates [74,75], there is evidence that pulsed lavage reduces the absolute risk of SSIs by 10.9% compared with standard irrigation [74].

Evidence regarding the most effective antiseptic remains limited. Current research indicates that surfactants and antibiotics should generally be avoided in irrigation solutions [75]. Most existing studies in orthopaedic surgery are retrospective, highlighting the need for well-structured prospective randomised controlled trials (RCTs) to provide clearer, evidence-based guidance [76].

Normothermia and Oxygenation

Peri-operative hypothermia: Peri-operative hypothermia, usually defined as a temperature <36.0°C during the peri-operative period, can result from anaesthesia-induced thermoregulatory inhibition combined with exposure to a cold operating-room environment. It is estimated that peri-operative hypothermia occurs in 50%-70% of patients undergoing major surgery [77].

Hypothermia may increase SSI risk and delay wound healing [78]. A 2014 retrospective cohort study suggested hypothermia is not an independent SSI risk factor; however, in patients with elevated HbA1c (poor glycaemic control), the incidence of SSIs doubled when the patient was hypothermic peri-operatively [79].

Supplemental oxygenation: Supplementing patients with a high fraction of inhaled oxygen (FiO₂) during the peri-operative phase may decrease SSIs. A 2022 systematic review and meta-analysis in colorectal surgery showed that inspired oxygen fractions ≥80% reduced SSIs by up to 27% [2]. A WHO study including 15 randomised controlled trials reported benefit with increased peri-operative FiO₂ 80% vs. standard FiO₂ (30%-35%) [80]. Despite this, NICE does not specifically advise high-flow oxygen; rather, it recommends maintaining oxygen saturations >95% [19].

Wound Closure

Suture types vary in size and composition and are used to close joint capsules, deep fascia, subcutaneous tissue and skin. Monofilament sutures have been considered preferable due to less surface area for pathogen harbourage. To prevent microbial colonisation, sutures with antimicrobial properties were developed. Recent research shows promising results for triclosan-coated sutures. NICE reported that triclosan-coated sutures reduced SSIs compared with standard absorbable sutures [81], with clear evidence of benefit in paediatric surgery.

Although triclosan-coated sutures cost more, savings from reduced SSIs outweigh the additional expense. Based on low- to medium-quality evidence, the WHO [13] and CDC [1] recommend antimicrobial-coated sutures, as potential SSI reduction outweighs associated risks.

Postoperative strategies

Wound Dressing

Negative-pressure wound therapy (NPWT), also known as vacuum-assisted closure, is a specialised dressing used to promote healing of surgical wounds. It applies sub-atmospheric pressure to the wound and is sealed with a suction pump and drainage system [82]. PICO™ (Smith & Nephew plc, Watford, England) is a type of NPWT that has been shown to decrease the incidence of SSI [83]. NICE also recommends PICO dressings, noting fewer SSIs and seromas vs. standard dressings [84].

A randomised controlled trial of 220 patients undergoing total hip and knee arthroplasty showed that NPWT reduced SSIs and prevented prolonged hospital stays [85]. Though not orthopaedic, NPWT showed a 68.8% reduction in SSIs after pancreaticoduodenectomy, further supporting its efficacy [86]. Regular monitoring and timely dressing changes help detect early signs of infection and maintain a cleaner wound environment [12].

Early Mobilisation

Early post-operative mobilisation is associated with shorter hospital stay and fewer major and minor postoperative complications [87,88]. In patients undergoing hip replacement, early mobilisation improved immune function and reduced SSI risk [89]. Another study showed SSI risk was 120% higher in patients who did not mobilise within 36 hours of surgery [9].

## Conclusions

Preventing SSIs in orthopaedic surgery necessitates a comprehensive, multidisciplinary approach to achieving optimised preoperative preparation, intra-operative techniques, and vigilant post-operative care and monitoring. Evidence supports the efficacy of patient risk factor modification, preoperative antibiotics prophylaxis, antiseptic measures, oxygenation, maintenance of normothermia and the use of negative pressure wound management. Ongoing research and adherence to clinical guidelines are essential to adapt to emerging challenges such as antibiotic resistance. Implementing evidence-based practices consistently across healthcare settings will be critical in reducing the burden of SSIs and improving patient outcomes in orthopaedic surgery.
